# Effects of Collagen Peptides Derived from Perch Scale Hydrolysates on the Physiological Activity of Osteoblasts

**DOI:** 10.3390/ijms27156777

**Published:** 2026-07-29

**Authors:** Chih-Ping Hsu, Hsiang Chang, Ling-Ni Chen, Mao-Hsiang Lee, Chih-Cheng Lin

**Affiliations:** 1 Department of Medical Laboratory Science and Biotechnology, Yuanpei University of Medical Technology, Hsinchu 300102, Taiwan; hsucp@mail.ypu.edu.tw; 2 Department of Biotechnology and Pharmaceutical Technology, Yuanpei University of Medical Technology, Hsinchu 300102, Taiwan; hchang@mail.ypu.edu.tw; 3 Research and Development Institute, Anyong Biotechnology Inc., Kaohsiung 827012, Taiwansam.lee@topco-global.com (M.-H.L.)

**Keywords:** bone, collagen peptides, hydrolysate, osteoblasts, osteogenesis, perch

## Abstract

A plethora of studies have demonstrated the bioactive properties of collagen peptides, including the promotion of wound healing and bone health. Research has demonstrated that these effects are attributable to their elevated biocompatibility and signaling capabilities. The present study investigated the effects of collagen peptides produced by enzymatic hydrolysis of perch scales on the induction of extracellular matrix formation and osteogenesis. The findings demonstrated that a combination of pre-heating and enzymatic hydrolysis resulted in the optimal peptide yield, with 94.9% of the peptides exhibiting a molecular weight below 1200 Daltons and containing elevated levels of hydroxyproline. The addition of perch scale collagen peptides to fibroblasts 890510-01F ATIT has been demonstrated to effectively induce the production of type I procollagen and fibronectin, a protein associated with the osteoblast phenotype and osteoblast differentiation. It is also worthy of note that experiments conducted using MC3T3-E1 osteoblasts indicate that collagen peptides significantly increased alkaline phosphatase activity by a factor of 1.46. This study demonstrates that collagen peptides derived from perch scales are enriched in bioactive peptides containing C-terminal tyrosine residues, including DYPRNHY and DPYNRHY. These findings provide a scientific basis for the future development of perch scale-derived collagen peptides as dietary supplements or functional food ingredients for promoting bone health.

## 1. Introduction

Collagen is the most significant extracellular protein in animal bodies, playing a crucial role in tissue structure. As demonstrated in the relevant literature, the phenomenon in question has been shown to have two main functions. Firstly, it has been demonstrated to maintain the structural integrity and strength of tissues [1]. Secondly, it has been demonstrated to provide essential mechanical protection for various tissues and organs [2,3]. Collagen, a protein derived from animal tissues, has a wide range of industrial applications, including in the leather, cosmetics, and pharmaceutical sectors. Furthermore, substantial quantities of collagen are utilized in the production of gelatin, which possesses a range of functional properties. These include its capacity to function as a gel, emulsifier, thickener, stabilizer, and colloid [4]. Collagen peptides are produced through enzymatic hydrolysis or microbial fermentation and consist of sequences of two to 20 amino acids. They can help to prevent chronic diseases and promote health by regulating and improving physiological functions such as lowering blood pressure, providing antioxidant effects, reducing cholesterol and exerting anticoagulant, calcium-binding, anti-obesity and anti-diabetic activities, as well as performing immunomodulatory functions [5,6,7]. Many different types of collagen peptides have been shown to regulate processes such as cell proliferation, migration, and apoptosis, and to inhibit angiogenesis [8].

The onset of bone health issues is often associated with the development of osteoporosis. This is due to an imbalance in the activity of osteoblasts and osteoclasts within the lacunae of the bone matrix, which leads to disorders of bone metabolism that subsequently affect joint stability and function [9]. Low osteoblast activity or overly active osteoclasts result in resorption exceeding bone formation, leading to bone loss and deterioration of the bone microstructure [10]. Post-translational modifications of collagen appear to play an indispensable role in the process of matrix mineralization, as well as influencing the mineralization density and crystalline structure of tissues [11]. Dietary type I collagen and its hydrolysates can increase osteoblast activity and aid bone remodeling [12,13]. Collagen or collagen hydrolysate intake is beneficial for bone health, helping to prevent osteoporosis and promote bone repair [14,15,16]. Studies have confirmed that shark skin gelatin improves type I collagen and bone mineral density in the bones of ovariectomised rats and demonstrated that collagen hydrolysates strengthen bone mineral density and bone strength in ovariectomised mice [17]. Furthermore, studies have observed an increase in organic matter content in bones following long-term consumption of collagen hydrolysate [18]. Collagen oligopeptides hydrolyzed from wild chum salmon (*Oncorhynchus keta*) with a molecular weight distribution of 100–860 Daltons (Da) (85.86% of which falls within the 300–860 Da range) were found to promote long bone development in growing rats. The study inferred that this effect is achieved by enhancing osteoblast activity and that this may be due to specific oligopeptide sequences rather than the total amino acid content [19]. At low concentrations, fish collagen peptides can increase the expression of genes related to osteoblast proliferation, differentiation and collagen-modifying enzymes. They can also improve bone collagen quality and promote mineralization via the mitogen-activated protein kinase and Smad signaling pathways [20]. It has been demonstrated that collagen hydrolysates isolated from porcine gelatin can modulate the processes of bone formation and resorption in cases of postmenopausal osteoporosis [21]. Low-molecular-weight (<1 kDa) porcine peptides, predominantly comprising tripeptides, have been shown to inhibit apoptosis and promote osteoblast proliferation and differentiation by activating the PI3K/Akt pathway [22]. Meanwhile, collagen peptides derived from milkfish scales have been shown to promote osteoblast synthesis while reducing RANKL-induced osteoclast activity. Among these peptides, a 1 kDa peptide has also been found to inhibit bone density loss in osteoporotic mice [23]. These results suggest that peptides of 1 kDa or smaller may exhibit greater osteogenic activity; however, further research is warranted regarding the molecular weight range and peptide sequences.

During the processing of fish, 20–80% of by-products, including skin, bones, heads and offal, are either wasted due to improper handling or downgraded for use as animal feed. These by-products are rich in bioactive nitrogen-containing compounds and proteins, with fish scales being particularly abundant in collagen. Recovering, reusing and developing these by-products into functional peptide raw materials would not only reduce environmental pollution, but also enhance value, promote resource recycling and drive the development of a green economy [24]. However, collagen in natural fish scales is tightly bound to hydroxyapatite, making extraction impossible using water or other solvents. Therefore, methods such as the use of acidic or alkaline reagents or enzymatic hydrolysis must be employed to extract collagen peptides from fish scales [25,26]. Chemical processing typically involves long production cycles and environmental pollution, whereas enzymatic processing offers faster reaction rates and generates less waste. Previous studies have found that collagen peptides derived from perch side streams through enzymatic hydrolysis can promote the production of type I procollagen, fibronectin, and hyaluronan in fibroblasts, thereby benefiting skin health and wound healing [27]. While some studies have examined the relationship between fish by-products and their hydrolysates in relation to bone health, few have analyzed the composition and characteristics of collagen peptides produced by the enzymatic treatment of perch scales or investigated their potential as dietary supplements to promote bone health. Therefore, this study aims to develop a method for directly extracting collagen peptides from perch scales, investigating the characteristics of these peptides and their osteogenic activity.

## 2. Results

### 2.1. Production of Collagen Peptides from Perch Scale

The amounts of free peptides obtained from perch scales under different treatment conditions are shown in Table 1. The yield of peptides obtained through heat treatment was found to be minimal; even when the temperature was elevated to 120 °C, a mere 5.17 mg/mL of free peptides were produced. Enzymatic hydrolysis using Protamex^®^ yielded 11.78 mg/mL of peptides, while treatment with a combination of three enzymes (Protamex^®^, Alcalase^®^ and Flavourzyme^®^) increased this to 13.48 mg/mL. In accordance with the findings of other studies [28], it is evident that the combination of enzymes is more efficacious than the use of a single one. As demonstrated in Table 1, the application of heat treatment subsequent to combined enzymatic hydrolysis has been shown to result in a substantial increase in the amount of free peptides, reaching a concentration of 31.15 mg/mL. The findings indicate that a higher peptide recovery rate can be attained by initially subjecting the material to heat treatment and subsequently employing enzymatic hydrolysis [26]. Pure perch scales are processed using an acid-washing method to remove the hydroxyapatite. However, since the collagen in fish scales has a robust protein structure, it must first undergo high-temperature treatment to break down its triple-helix structure. The collagen is then enzymatically hydrolyzed to break it down into smaller peptide molecules, resulting in a pure fish scale collagen peptide solution. The maximum yield of perch scale peptide product obtained under these conditions is designated as PS.

### 2.2. The Profile of PS

#### 2.2.1. Molecular Weight Distribution

The molecular weight distribution of PS was analyzed using gel permeation chromatography. As demonstrated in Table 2, a mere 1.3% of PS has a molecular weight greater than 2300 Da, while 94.9% has a molecular weight less than 1200 Da, and 73.58% has a molecular weight less than 400 Da. It is estimated that the average molecular weight of PS is approximately 383 Da. This is indicative of peptides which are composed of approximately 3 to 4 amino acids. The bioavailability of small-molecule peptides, such as tripeptides and tetrapeptides, which typically possess molecular weights ranging from 300 to 500 Da, has been demonstrated to be excellent [29]. These peptides have the capacity to traverse the intestinal barrier, thereby entering the bloodstream [30]. In the context of bone and joint health, they fulfil a dual role as both messengers and building blocks.

#### 2.2.2. Amino Acid Composition

It is evident from a numerous studies that the collagen obtained from fish scales is likely to be type I collagen [31,32,33]. As illustrated in Table 3, the amino acid composition of PS exhibits notable variations, with glycine content reaching up to 2120 mg/100 g. When the contents of glycine, proline, and hydroxyproline—the amino acids that make up collagen—are combined, they account for nearly 44.6% of the total amino acid content. Consequently, it can be concluded that PS can be regarded as a collagen peptide. As established in our preceding research [27], perch meat has been found to contain 599 mg/100 g of hydroxyproline, which equates to approximately 4792 mg/100 g of collagen. However, the perch scale peptides produced in this study contain 908 mg/100 g of hydroxyproline, which is estimated to contain nearly 7264 mg/100 g of collagen. The results of this study suggest that perch scales contain a higher concentration of collagen than perch meat. In summary, the process of manufacturing involves the application of heat treatment in conjunction with enzymatic hydrolysis. This method ensures the effective extraction of collagen, consequently producing collagen peptides with enhanced bioavailability.

#### 2.2.3. Peptide Sequence

The results of the peptide sequence analysis of PS presented in Table 4 indicate that the sequence DYPRNHY accounts for 20.56%, and the sequence DPYNRHY accounts for 11.11%. The remaining sequences represented a proportion of less than 6.41%. It is noteworthy that these sequences all feature a Y (tyrosine) at either the N-terminal or the C-terminal. Tyrosine possesses a phenolic hydroxyl group, denoted as -OH, which facilitates interaction with ions present on the surface of bone minerals via hydrogen bonds when positioned at the end of the sequence. In the event of Y being located at the head or tail, it has been demonstrated to facilitate a more robust attachment of the peptide chain to the bone matrix [34,35]. The bone-targeting property of the peptide facilitates its localised retention within bone tissue, thereby concentrating therapeutic signals at the target site while reducing rapid systemic clearance. Aromatic residues, such as tyrosine, are recognized as substrate targets for specific endopeptidases [36]. However, spatial constraints derived from the specific sequence architecture and terminal residues have the capacity to restrict protease accessibility to susceptibility sites [37]. Consequently, peptides comprising a terminal Y (e.g., DYPRNHY and DPYNRHY) exhibit enhanced metabolic stability following their entry into the bone marrow microenvironment, thereby sustaining long-term osteogenic signaling.

### 2.3. The Bioactive Properties of PS Related to Bone Health

In order to comprehensively elucidate the multifaceted biological activity of PS without the influence of confounding variables, this study strategically selected two highly specialized cell lines, 890510-01F fibroblasts and MC3T3-E1 osteoblast precursors, to represent different functional stages of tissue development. The 890510-01F Fibroblast is characterized by its inherent capacity for high levels of extracellular matrix synthesis, thus serving as a standard model for the evaluation of the basal synthetic capacity of structural proteins, including procollagen, fibronectin and hyaluronan. Conversely, the MC3T3-E1 line has been specifically adapted for the study of the biochemical transitions of osteogenesis and mineralization, thus rendering it the ideal specific model for tracking ALP induction.

#### 2.3.1. PS-Induced Extracellular Matrix Production in 890510-01F Fibroblasts

**Figure 1:**
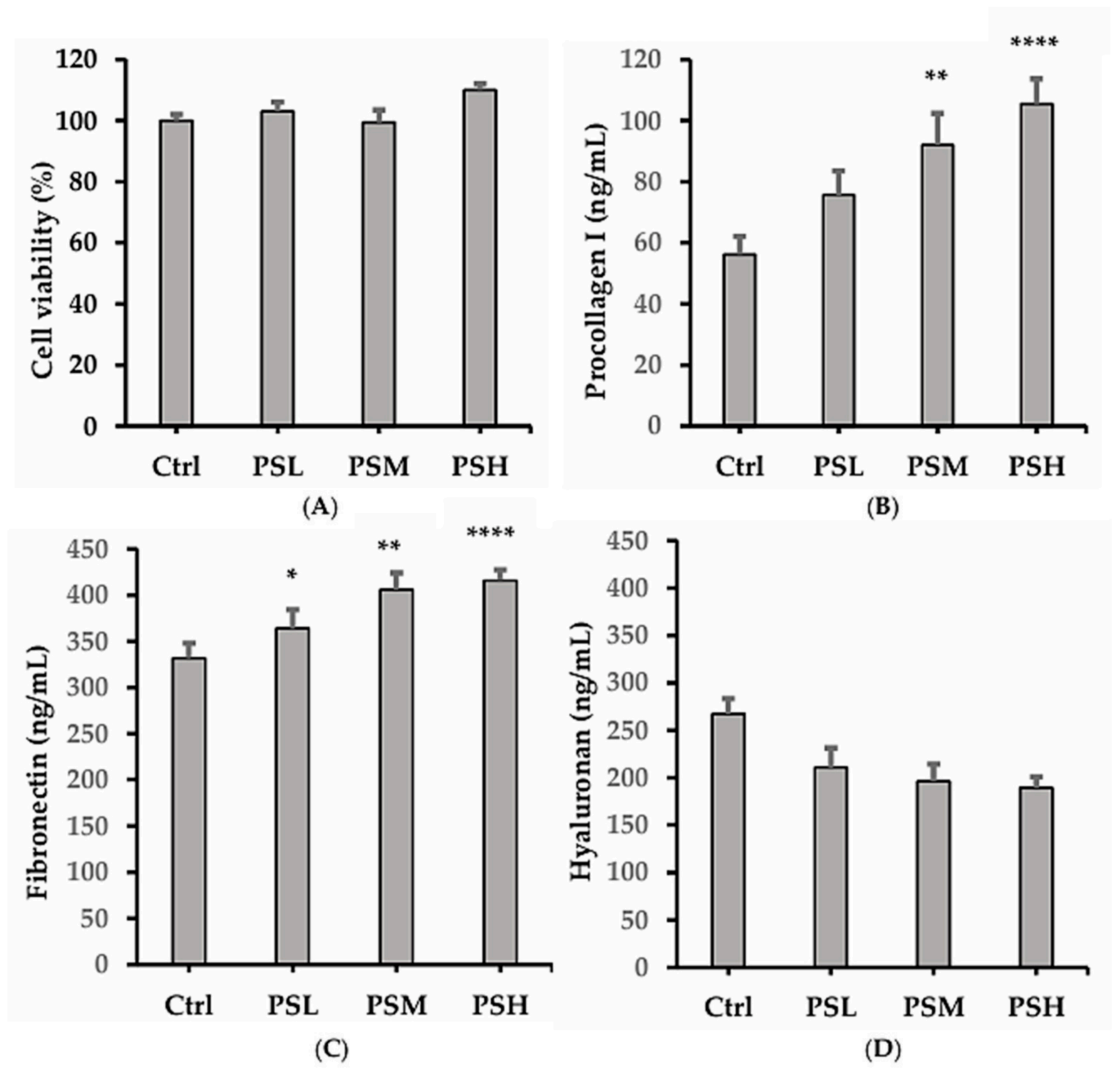
Effects of perch scale peptide at various concentrations on human dermal 890510-01F fibroblasts after 48 h of incubation. (**A**) Cell viability, and the production levels of (**B**) procollagen I, (**C**) fibronectin, and (**D**) hyaluronan. PSL, PSM, and PSH represent peptides added at concentrations of 1.33, 2.66, and 5.31 mg/mL, respectively. Statistically significant differences were analyzed using one-way ANOVA, where * *p* < 0.05, ** *p* < 0.01, and **** *p* < 0.0001 indicate varying levels of significance.

The PS stock solution, prepared via thermal and enzymatic hydrolysis, was diluted to various concentrations and administered to human dermal fibroblasts (890510-01F ATIT) for a 48-h incubation. The treatment concentrations were selected based on preliminary cytotoxicity screening, where 5.31 mg/mL was identified as the highest non-cytotoxic concentration (>90% cell viability), followed by serial two-fold dilutions to evaluate dose-dependent responses. The results demonstrated that none of the tested concentrations—1.33 mg/mL (PSL), 2.66 mg/mL (PSM), and 5.31 mg/mL (PSH)—exhibited signs of toxicity (Figure 1A). Notably, PS treatment significantly up-regulated the secretion of both procollagen I (Figure 1B) and fibronectin (Figure 1C) in a clear dose-dependent manner, with higher concentrations yielding more pronounced production. Regarding hyaluronan synthesis (Figure 1D), although a slight downward trend was observed following PS supplementation, this reduction did not reach statistical significance. The results of this study indicate that PS specifically promotes the expression of procollagen I and fibronectin, but has no significant effect on hyaluronan production. This finding suggests the possibility that the role of PS is primarily responsible for the structural remodeling of the extracellular matrix and the bone mineralization pathways rather than for the synthesis of general mucopolysaccharides. In consequence, the efficacy of PS in bone health is primarily attributable to its ability to strengthen the bone matrix.

#### 2.3.2. Effects of PS on Alkaline Phosphatase Activity

**Figure 2:**
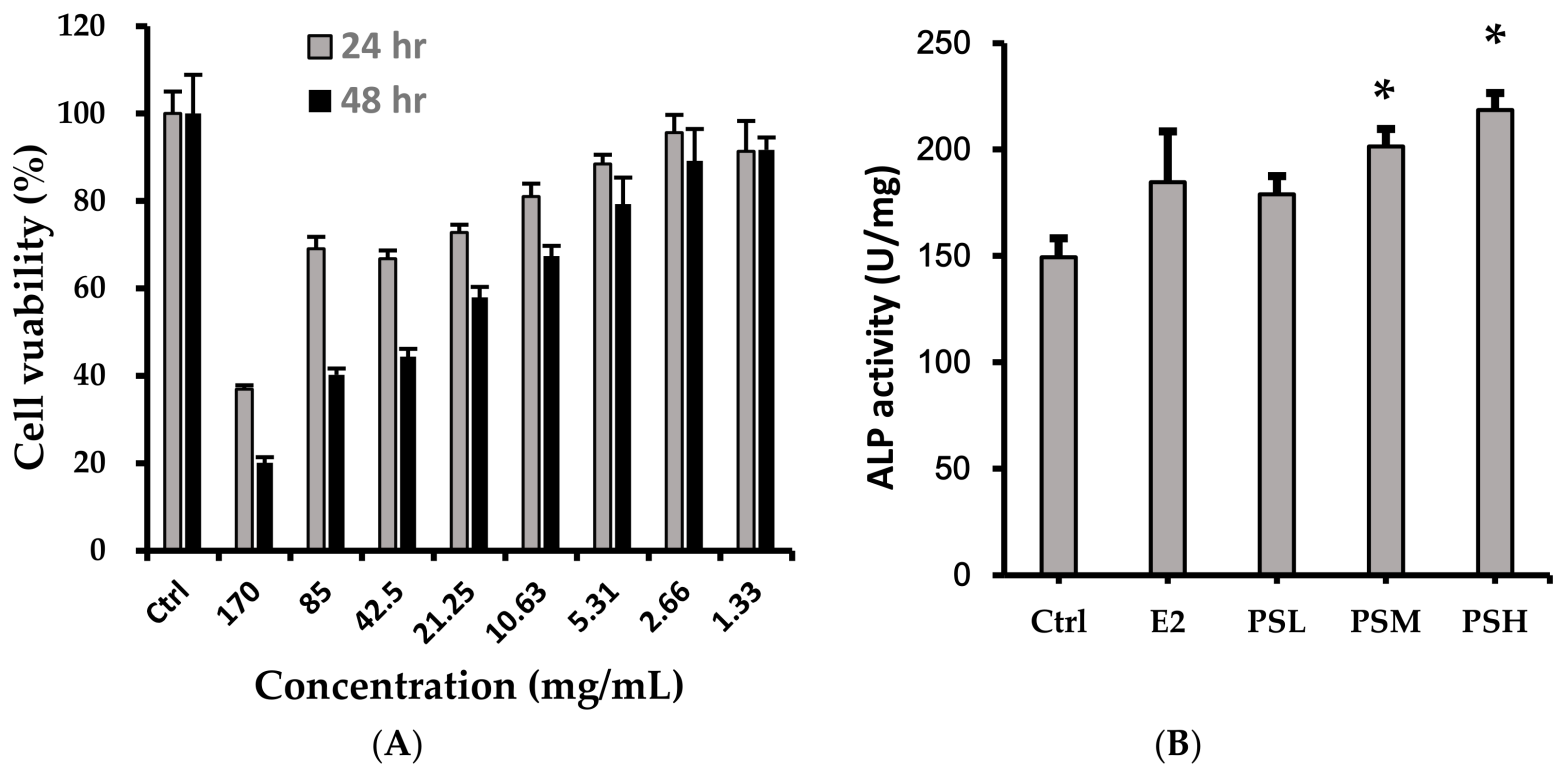
Effects of PS on MC3T3-E1 cells across different incubation time points. (**A**) Cell viability determined after 24 h and 48 h of treatment. (**B**) Alkaline phosphatase (ALP) activity measured after 7 days of treatment. Cells in the positive control group (E2) were treated with 100 nM beta-estradiol, while those in the PSL, PSM, and PSH groups were treated with 1.33, 2.66, and 5.31 mg/mL of hydrolysate, respectively. Asterisks (*) indicate significant differences relative to the control group (*p* < 0.05).

To determine the effects of PS on bone health, we used a pre-osteoblast cell line (MC3T3-E1) derived from mouse calvaria for further observation and investigation. As shown in Figure 2A, no cytotoxicity was observed at concentrations of 5.31 mg/mL or lower. Alkaline phosphatase (ALP) is a key marker for the differentiation of MC3T3-E1 cells from pre-osteoblasts into mature osteoblasts. PS significantly increases ALP activity, with higher concentrations resulting in greater activity (Figure 2B). While a stable organic scaffold is imperative, the transformation into functional bone tissue is contingent upon an active mineralization process driven by osteoblasts. In this study, MC3T3-E1 pre-osteoblasts were exposed to PS, resulting in a significant increase in ALP activity. It has been demonstrated that ALP catalyzes the hydrolysis of extracellular pyrophosphate, a substance which has been shown to inhibit calcification, into inorganic phosphate. The resulting rise in local phosphate ion concentration reacts with surrounding calcium ions (Ca^2+^), promoting the precipitation of hydroxyapatite crystals onto the preformed collagen-fibronectin network. Consequently, the augmentation in ALP activity in MC3T3-E1 cells underscores the notion that these collagen peptides proactively instigate a maturation sequence in pre-osteoblasts, thereby facilitating their conversion to a state characterized by stimulates early-stage osteoblast activity.

## 3. Discussion

The findings of the study demonstrate that preheating, followed by hydrolysis with a combination of enzymes, is an effective method for producing collagen peptides (PS) from perch scales. Molecular weight distribution analysis demonstrated that the PS fraction is predominantly composed of low-molecular-weight compounds. It is noteworthy that 94.90% of the total peptides had a molecular weight below 1200 Da, with small-molecule oligopeptides (<400 Da) accounting for 73.58% of the total distribution. The significant abundance of these highly biocompatible low-molecular-weight peptides (383 Da) is extremely beneficial for gastrointestinal absorption, as dipeptides and tripeptides can be efficiently transported into the bloodstream via transport proteins, potentially serving as signaling molecules for bone health.

Research has demonstrated that short peptide fragments, abundant in tyrosine (Y) and histidine (H), possess the capacity to stimulate the transcription of relevant genes within the cell nucleus. This stimulation is achieved through the upregulation of intracellular TGF-β/Smad or MAPK/ERK signaling pathways, thereby resulting in a substantial augmentation in the secretion of procollagen I and fibronectin [38]. This study corroborates the hypothesis that bioactive short peptides ending in Y or HY, which are abundant in PS, act on 890510-01F cells to significantly activate and proliferate key components of the extracellular matrix (ECM), particularly by effectively promoting the production of procollagen I and fibronectin. In the molecular and biochemical mechanisms of tissue repair and bone matrix formation, fibronectin functions as a bridge for cell adhesion and migration, providing anchoring signals through integrin receptors on the cell surface to guide directed cell aggregation. Procollagen I and fibronectin, meanwhile, serves as the primary fibrous building block for constructing the rigid scaffold of the organic bone matrix.

The active short peptides contained in the PS (e.g., DYPRNHY and DPYNRHY) used are characterized by a secondary structure that integrates key conserved residues with strong chelating properties and high osteogenic activity. Specifically, the sequence comprises aspartic acid (D) at the N-terminus, histidine (H) in the middle, and tyrosine (Y) at the C-terminus. As demonstrated in the relevant studies, the imidazole group of histidine, the phenolic ring of tyrosine, and the carboxyl group of aspartic acid have been identified as the core sites in the peptides responsible for forming coordination bonds with metal/calcium ions [39,40]. This provides a solid evidence base for calcium chelation and nucleation guidance. Furthermore, the C-termini of these peptides in PS precisely expose the tyrosine (Y) residue, a structural feature that offers a significant advantage in structural recognition due to its high similarity to the core fragment of the endogenous osteogenic growth peptide. Short peptides containing such C-terminal tyrosine residues have been shown to bind to receptors on the surface of osteoblasts with effectiveness, resulting in a significant upregulation of ALP activity and promoting osteoblast maturation. This effect is achieved through the activation of the downstream MAPK/ERK signaling pathway [34]. Concurrently, the tyrosine-rich short peptide structure has been demonstrated to regulate the deposition and growth of hydroxyapatite crystals during biomineralization with efficiency [41]. The synergistic interaction of these biochemical mechanisms forms the theoretical basis for how collagen short peptides in PS promote bone mineralization and osteoblast differentiation. In the present study, treatment with peptides containing terminal tyrosine led to a significant elevation in ALP activity at Day 7 in MC3T3-E1 cells. As ALP serves as a primary functional marker for early osteogenic commitment, these results indicate that the peptides effectively stimulate early osteoblastic differentiation. However, it is acknowledged that ALP activity alone is insufficient to demonstrate full-stage osteogenesis. The process of complete functional maturation is characterised by the occurrence of late-stage extracellular matrix mineralization and subsequent downstream gene activation (including Runx2, OCN, and OPN). Whilst the present findings provide considerable support for the enhancement of osteoblast activity, further studies evaluating matrix mineralization assays (e.g., Alizarin Red S staining) and protein-level transcriptional profiling will be valuable in order to fully elucidate the long-term osteogenic potential of these peptides.

These findings demonstrate that the short peptides in PS, with an average molecular weight of 383 Da, exhibit excellent activity in 890510-01F fibroblasts and MC3T3-E1 osteoblasts. Furthermore, they confirm that the Y structure at both ends of the peptide and the H structure in the middle possess a dual regulatory mechanism for bone strengthening and tissue repair, involving the promotion of matrix protein synthesis and the guidance of calcium salt mineralization. However, the crude PS fraction was evaluated in its totality, without the implementation of subsequent activity-guided purification. While the identification of individual bioactive sequences is pivotal to comprehending precise molecular targets, complex peptide hydrolysates frequently function through multi-component synergy, whereby different peptides simultaneously exert influence on complementary cellular pathways [42,43]. Moreover, evaluating the complete fraction is congruent with practical industrial applications, wherein whole functional hydrolysates provide scalable and cost-effective solutions. Nevertheless, we acknowledge that activity-guided fractionation and detailed peptidomic profiling remain essential next steps to isolate key active sequences and decipher their individual mechanistic contributions.

Although our in vitro findings demonstrate that fish-scale collagen peptides possess significant osteogenic and mineralization-promoting activities in osteoblasts, several important questions remain to be addressed. Future studies should validate these findings in vivo using bone defect or osteoporosis animal models to evaluate their bioavailability, pharmacokinetic properties, tissue distribution, and therapeutic efficacy. In addition, comprehensive structural characterization combined with transcriptomic and proteomic analyses will be essential to elucidate the molecular mechanisms underlying their biological activities, including interactions between specific bioactive peptide sequences and cell-surface receptors (e.g., integrins), as well as the downstream signaling pathways and gene regulatory networks involved in osteogenesis. Such mechanistic insights will provide a strong scientific foundation for the development of fish-scale collagen peptides as functional food ingredients, nutraceuticals, or therapeutic agents for the prevention and management of bone-related disorders.

## 4. Materials and Methods

### 4.1. Perch Scale Hydrolysate Preparation

Fish scales were collected from adult perch (*Lates calcarifer*) with a weight exceeding 1.2 kg. These were provided by Anyong Biotechnology, Inc. (Kaohsiung, Taiwan). These perch were cultivated under a contract ecological aquaculture initiative and reared for a period more than 10 months using standard freshwater and brackish water aquaculture practices. The pure fish scales were obtained by removing the scales from the bodies of the fish using a drum-type descaling machine. These were then soaked in a 2% citric acid solution, which was replaced twice to remove the hydroxyapatite, before being rinsed with clean water to produce the purified scales.

These scales were then mixed with water at a ratio of 1:3 (*w*/*v*) and hydrolyzed under the operating conditions listed in Table 1. Heat treatment was performed at 60 °C or 120 °C for one or two hours. After cooling, a single enzyme or a combination of three enzymes was added for hydrolysis at 60 °C. A temperature of 60 °C was selected because it provides optimal protease activity while exceeding the thermal denaturation threshold of fish collagen. This promotes thermal unfolding of the collagen triple helix, thereby exposing proteolytic cleavage sites, reducing reaction medium viscosity, and suppressing microbial growth during prolonged incubation. These three enzymes were a protease complex (Protamex^®^), an alkaline protease (Alcalase^®^) and an exo-protease (Flavourzyme^®^), all of which were purchased from Novonesis Japan Ltd. (Chiba, Japan). Finally, the mixture was treated at 100 °C for 10 min to inactivate the enzymes. After cooling, the fish scale hydrolysate was filtered through a 300-mesh stainless steel sieve. The resulting filtrate was then freeze-dried for storage and subsequent experimental analysis.

### 4.2. Determination of Free Peptide Content

The method for determining free peptide content was adapted from that of Church et al. (1983) with slight modifications [44]. After appropriate dilution of the fish scale hydrolysate, 50 μL of the diluted sample solution was added to 2 mL of o-phthaldialdehyde solution. The mixture was shaken to ensure uniformity and allowed to stand in the dark at room temperature for 2 min. The absorbance was then measured at 340 nm using a spectrophotometer (SP-UV1100, DLAB Scientific Inc., Beijing, China) to measure the absorbance at 340 nm. The peptide concentration was calculated using a standard calibration curve derived from Leu-Gly standard solutions, expressed in mg/mL. The highest yield of hydrolysate of perch scales is achieved under different hydrolysis conditions, which is hereinafter defined as PS.

### 4.3. Determination of Molecular Weight Distribution

The molecular weight distribution of the PS was analyzed using gel permeation chromatography (GPC). Prior to GPC analysis, a 5 mL sample was filtered through a 0.22 µm PES (polyethersulfone) filter to remove insoluble particles. The analytical system used a Viscotek GPC system (Malvern Instruments, Malvern, UK) comprising a pump (1122), an injector (7125i), a laser light scattering detector (270LS) and a refractive index detector (Shodex 71). This was paired with the OmniSEC 4.6 software system (Malvern Panalytical, Malvern, UK) for data acquisition and analysis. A GMPWxl column was used, with the column temperature maintained at 30 °C. The mobile phase consisted of a 0.05 M aqueous solution of NaNO_3_, with a flow rate of 0.8 mL/min. Each analysis lasted 30 min, with an injection volume of 100 µL. Both the laser light scattering detector and the refractive index detector were employed for detection during the analysis.

### 4.4. Determination of Amino Acid Compositions and Identification of Peptide Sequence

The amino acid composition of PS was analyzed using an amino acid analyser (Thermo Fisher Scientific, Inc., Waltham, MA, USA), in accordance with ISO 13903:2005 and the methods described in previous studies [27]. Peptide sequence identification was also performed using the LC/MS/MS analysis described in our previous studies [27].

### 4.5. Analytical Assays for Fibronectin, Procollagen I and Hyaluronan in Human Fibroblasts

Human dermal fibroblasts (890510-01F ATIT) were cultured to induce differentiation. The samples were incubated at 37 °C for 2 days. The supernatant was then collected, and the concentrations of fibronectin, type I procollagen, and hyaluronic acid were quantified using an enzyme-linked immunosorbent assay (ELISA) according to the method described in our previous study [27].

### 4.6. Analysis of the Effect on MC3T3-E1 Cell Differentiation and ALP Activity

The MC3T3-E1 cells were purchased from the European Collection of Authenticated Cell Cultures (ECACC). The cell culture medium used to culture the cells consisted of 89% Minimum Essential Medium α (MEMα) (Gibco, Grand Island, NY, USA), 10% Fetal Bovine Serum (Cytiva, Marlborough, MA, USA) and 1% 100× Antibiotic–Antimycotic Solution (Gibco, Grand Island, NY, USA). Place the cell cryovials in a 37 °C water bath until they are completely thawed. Transfer the cell suspension to an appropriate volume of culture medium, then place it in an incubator for cultivation at 37 °C, with 5% CO_2_ and 90% humidity. Once the cell density reaches at least 80%, proceed with cell passaging. During this process, remove the original culture medium and rinse the dish with phosphate-buffered saline (PBS) (Gibco, Grand Island, NY, USA). Then add trypsin to detach the cells and suspend them. Finally, transfer an appropriate number of cells to a container with fresh culture medium for further cultivation. PS was dissolved in cell culture medium at a concentration of 170 mg/mL and then titrated with 10 N NaOH (Apollo, New York, NY, USA) until the medium containing phenol red turned orange-red. To avoid inappropriate pH levels from affecting cell growth, pH measurements were taken before and after titration using a pH meter (Sartorius, Göttingen, Germany). PS was then diluted with cell culture medium to achieve concentrations of 1.33 mg/mL (PSL), 2.66 mg/mL (PSM) and 5.31 mg/mL (PSH). The culture medium containing PS must be used within 10 days of preparation. If this time limit is exceeded, a new batch must be prepared.

Following thawing, the cells were cultured for at least two passages before being seeded into a 96-well cell culture plate at a density of 3 × 10^3^ cells per well. Following an overnight incubation period, 100 μL of cell culture medium containing various concentrations of PS were added to each well of the plate. The cells were then cultured for either 24 or 48 h. Prepare the 3-(4,5-dimethylthiazol-2-yl)-2,5-diphenyltetrazolium bromide (MTT) reaction solution by mixing a PBS solution containing 5 mg/mL MTT with fresh cell culture medium in a 5:1 ratio. Next, remove the culture medium and add 120 μL of the MTT reaction solution to each well of the 96-well plate containing cells. After one hour of incubation, remove the culture medium and add 100 μL of dimethyl sulfoxide (DMSO) (Apollo, New York, NY, USA). After leaving to stand at 37 °C for 15 min, measure the absorbance at 570 nm using an Infinite 200 Pro microplate reader (Tecan, Männedorf, Switzerland). Calculate the cell survival rate using the following formula: Cell survival rate (%) = [A_sample_ − A_blank_]/[A_C_ − A_blank_] × 100, where A_sample_ is the absorbance value of the control or test group, A_blank_ is the absorbance value of the blank group, and A_C_ is the absorbance value of the control group.

Following thawing, the cells were cultured for at least two passages and then seeded at a density of 6 × 10^4^ cells/well into a 24-well cell culture plate. Following an overnight culture period, the medium was replaced with 1 mL of differentiation medium containing either 100 nM β-estradiol (E2) or different concentrations of PS. Replace the medium with fresh differentiation medium on days 3 and 6, then continue culturing until day 7. Remove the culture medium from each well of the 24-well plate and add 1 mL of 4 °C PBS on ice to rinse each well. After removing the PBS, add 200 μL of PBS containing 1% Triton X-100 to each well and leave to stand on ice for 30 min. Transfer the cell lysate to a 1.7 mL microcentrifuge tube and centrifuge at 4 °C and 20,360× *g* for one minute. Then, collect the supernatant for the alkaline phosphatase (ALP) activity assay and protein quantification. The alkaline phosphatase activity assay was performed according to the protocol of the Alkaline Phosphatase Assay Kit (Colorimetric) (Abcam, Waltham, MA, USA). Mix 20 μL of the cell lysate supernatant with 60 μL of ALP assay buffer. Then use an Infinite 200 Pro microplate reader to measure the absorbance at 405 nm and calculate the enzyme activity (U/mL). Protein concentration was determined using the Pierce™ BCA Protein Assay Kit (Thermo Scientific, Waltham, MA, USA). The cell lysate supernatant was used for protein quantification and the concentration (μg/mL) was calculated based on absorbance at 562 nm. ALP specific activity was calculated using the following formula and is expressed as ALP activity units per mg of protein: ALP specific activity (U/mg) = [ALP activity (U/mL)/protein concentration (μg/mL)] × 1000.

### 4.7. Statistical Analysis of Data

Each experiment was conducted in triplicate. The data were analyzed using Microsoft Excel, which is part of the Office suite, and the results are presented as the mean ± standard deviation (SD). Differences between groups were compared using the Dunnett test within a one-way ANOVA, which was performed using Minitab statistical software (Minitab 22). A *p*-value of less than 0.05 was considered statistically significant and a *p*-value of less than 0.001 was considered highly significant.

## 5. Conclusions

The results of this study establish the benefits of collagen peptides derived from hydrolyzed perch scales for bone health, as well as the comprehensive and interrelated mechanisms underlying these effects. These peptides have been shown to exert a synergistic dual effect, optimizing the organic architecture of bone by enriching the matrix with type I procollagen and fibronectin, whilst also activating mineralization mechanisms by upregulating ALP in osteoblasts. The dual action of the peptides under investigation highlights the potential of perch scale collagen peptides as a potent therapeutic agent or dietary supplement. This is in relation to maintenance of bone density as well as promotion of fracture healing.

## 6. Patents

For the perch scale cleaning, extraction, and filtration processes, refer in part to TWN Patent I883803.

## Figures and Tables

**Table 1:** Results of extraction or hydrolysis of purified perch scales under different processing conditions.

Processing Type	Thermal Condition		Enzyme Condition			Free Peptide ^1^ (mg/mL)
	Temp.	Time	Temp.	Time	Enzyme	
Thermal	60 °C	2 h	-	-	-	0.18 ± 0.05 ^e^
Thermal	120 °C	1 h	-	-	-	5.17 ± 0.06 ^d^
Enzyme hydrolysis	-	-	60 °C	4 h	Single Enzyme ^2^	11.78 ± 0.40 ^c^
Enzyme hydrolysis	-	-	60 °C	4 h	Complex enzyme ^3^	13.48 ± 0.30 ^b^
Thermal + enzyme hydrolysis ^4^	120 °C	1 h	60 °C	4 h	Complex enzyme ^3^	31.15 ± 0.40 ^a^

Mean ± standard deviation (n = 3). Different superscript letters in the same column indicate significant difference (*p* < 0.05) between samples. ^1^ Peptide content of the samples was determined using the OPA (o-Phthalaldehyde) spectrophotometric method. ^2^ Enzymatic hydrolysis was performed using a single enzyme, Protamex^®^. ^3^ Enzymatic hydrolysis was conducted using the complex enzyme combination as described in the materials and methods section. ^4^ Following the preparation method for perch scale collagen peptides, purified perch scales underwent heat treatment followed by enzymatic hydrolysis.

**Table 2:** Molecular Weight Distribution of Perch Scale hydrolysates.

Molecular Weight Distribution	Perch Scale Collagen Peptides
>2300 (Da)	1.30%
2300–1200 (Da)	3.80%
1199–580 (Da)	11.00%
579–240 (Da)	30.40%
239–189 (Da)	11.70%
<189 (Da)	41.80%
Peptide content < 1200 Da	94.90%
Peptide content < 400 Da	73.58%
Average Molecular Weight (Da)	383

**Table 3:** Concentration of Hydrolyzed Amino Acids in Perch hydrolysate.

Hydrolyzed Amino Acid Profiles	Concentration (mg/100 g)
Glycine	2120
Proline	1090
Alanine	977
Hydroxyproline	908
Glutamic acid	895
Arginine	718
Aspartic acid	526
Lysine	334
Threonine	259
Serine	257
Valine	237
Leucine	219
Phenylalanine	191
Methionine	190
Isoleucine	129
Histidine	108
Tyrosine	83
Total	6677

**Table 4:** Corresponding sequences identified by de novo sequencing of peptide samples and their proportions.

Peptide Sequence	Relative Abundance
DYPRNHY	20.56%
DPYNRHY	11.11%
YNKGF	6.41%
DRYY	5.56%
YPGGRHYD	4.72%
LYY	3.88%
PYY	3.58%
YDRY	3.36%
DPRGGYHY	3.17%
YPY	2.37%

## Data Availability

The original contributions presented in this study are included in the article. Further inquiries can be directed to the corresponding author.
